# IRAK3 Knockout and Wildtype THP-1 Monocytes as Models for Endotoxin Detection Assays and *Fusobacterium nucleatum* Bacteriophage FNU1 Cytokine Induction

**DOI:** 10.3390/ijms242015108

**Published:** 2023-10-12

**Authors:** Siti Saleha Binte Mohamed Yakob Adil, Mwila Kabwe, Cassandra Cianciarulo, Trang Hong Nguyen, Helen Irving, Joseph Tucci

**Affiliations:** 1 Department of Rural Clinical Sciences, La Trobe Rural Health School, La Trobe University, P.O. Box 199, Bendigo, VIC 3550, Australia; 2 La Trobe Institute for Molecular Science, La Trobe University, P.O. Box 199, Bendigo, VIC 3550, Australia

**Keywords:** antimicrobial resistance, bacteriophages, THP-1 monocytes, interleukin-1 receptor associated kinase-3 (IRAK-3), endotoxin, bacteriophage therapy

## Abstract

Microbial resistance to antibiotics poses a tremendous challenge. Bacteriophages may provide a useful alternative or adjunct to traditional antibiotics. To be used in therapy, bacteriophages need to be purified from endotoxins and tested for their effects on human immune cells. Interleukin-1 Receptor Associated Kinase-3 (IRAK3) is a negative regulator of inflammation and may play a role in the modulation of immune signalling upon bacteriophage exposure to immune cells. This study aimed to investigate the immune effects of crude and purified bacteriophage FNU1, a bacteriophage that targets the oral pathobiont *Fusobacterium nucleatum*, on wildtype and IRAK3 knockout THP-1 monocytic cell lines. The IRAK3 knockout cell line was also used to develop a novel endotoxin detection assay. Exposure to crude FNU1 increased the production of pro-inflammatory cytokines (Tumour necrosis factor – alpha (TNF-α) and Interleukin 6 (IL-6)) compared to purified FNU1 in wildtype and IRAK3 knockout THP-1 monocytes. In the IRAK3 knockout THP-1 cells, exposure to crude FNU1 induced a higher immune response than the wildtype monocytes, supporting the suggestion that the inhibitory protein IRAK3 regulates reactions to endotoxins and impurities in bacteriophage preparations. Finally, the novel endotoxin detection assay generated here provides a robust and accurate method for determining endotoxin concentrations.

## 1. Introduction

*Fusobacterium nucleatum* is an anaerobic Gram-negative bacterium found in the oral cavity and represents an important species in dysbiotic community development in periodontal plaques [1]. It is also associated with adverse pregnancy outcomes, and chronic diseases such as cardiovascular disease, rheumatoid arthritis, respiratory tract infections, Lemierre’s syndrome, Alzheimer’s disease, gastrointestinal disorders and cancers [2,3,4,5,6].

Antimicrobial resistance has become a major medical problem worldwide and is considered an overlooked pandemic [7]. Due to increasing worldwide trends of antibiotic-resistant bacterial infections, bacteriophages are being regarded as potential alternatives and adjuncts in assisting the treatment of these infections. They are predatory viruses found in environments where bacteria grow [8], and almost exclusively target a specific bacterium or a few strains within a particular species [9]. As such, they potentially offer precision antimicrobial therapy, as opposed to antibiotics, which have a much broader spectrum, and therefore, destroy not only intended pathogens but many other bacteria in the human microbiome that may contribute to human health [10,11,12]. Bacteriophages have also shown the capacity to kill antibiotic-resistant bacteria [13], and are capable of degrading biofilms of pathogenic strains [14]. The *F. nucleatum* bacteriophage FNU1 was the first fully characterised bacteriophage reported to target *F. nucleatum* specifically and degrade its biofilms [15]. Bacteriophage FNU1 has recently been classified as a new genus because of its genetic novelty [16].

One of the main issues in the application of bacteriophages in therapy is the question of their safety, and how they may affect the mammalian immune response. When isolated from bacterial populations, bacteriophage preparations may contain endotoxins such as lipopolysaccharides (LPS) [17], which can activate the Toll-like receptor 4 (TLR4) signalling pathway and inflammation [18,19]. TLR4 is the main driver that regulates innate immune response against bacterial infections [20]. Specifically, TLR4s recognise LPS in Gram-negative bacteria, which then induces the production of proinflammatory cytokines such as tumour necrosis factor -alpha (TNF-α) and interleukin 6 (IL-6). As a modulator of inflammation, interleukin-1 receptor associated kinase 3 (IRAK-3), has a negative effect on TLR4 signaling [21,22] and this is in part via the generation of Guanosine 3′,5′-cyclic monophosphate (cGMP) [23,24,25]. The precise effect of bacteriophages on the human immune system is unclear, and the role of IRAK3 has not been studied in this context. Bacteriophages have been reported to have diverse effects on immune cells, through the production of either anti or proinflammatory cytokines [26,27,28,29,30]. Further, while clinical trials have shown the safety and efficacy of bacteriophage therapy [31,32], there have been associations with adverse immunological events such as cytokine storm [33,34] and poor efficacy due to neutralising antibodies, despite purification of endotoxin in the bacteriophage preparation [33].

Strict regulations provided by bodies such as the Australian Therapeutic Goods Administration, European Medicines Agency and the United States Food and Drug Administration govern the maximal level of endotoxins for parenteral applications of pharmaceutical and biological therapeutics to prevent adverse events. The threshold pyrogenic dose of endotoxins is set at 500 pg of endotoxins per kg of body weight per hour, (which is defined as five endotoxin units (EU)) for non-intrathecal administration and 20 pg of endotoxins per kg of body weight per hour (0.2 EU) for intrathecal administration [35]. Therefore, purification of bacteriophages and measurement of EU units of the final preparation is important for their safe delivery in therapy. The aims of this study were to investigate the effects of the *F. nucleatum* bacteriophage FNU1 on wildtype and IRAK3 knockout THP-1 monocytic cell lines. The IRAK3 knockout cells, generated by our group, were used here as they have been shown to be more reactive toward LPS [25], and as such, provide a useful study model of immune dysfunction in humans. Crude FNU1, isolated directly from bacterial hosts, and purified FNU1, treated to remove endotoxins, were tested on these immune cells, which were assessed for production of the pro-inflammatory cytokines IL-6, TNF-α and the anti-inflammatory interleukin 10 (IL-10). Finally, the concentration of endotoxin present in crude and purified FNU1 was determined by a novel assay developed as part of this work.

## 2. Results

### 2.1. Effects of FNU1 on Cytokine Production in Wildtype and IRAK3 Knockout THP-1 Cells

**Figure 1:**
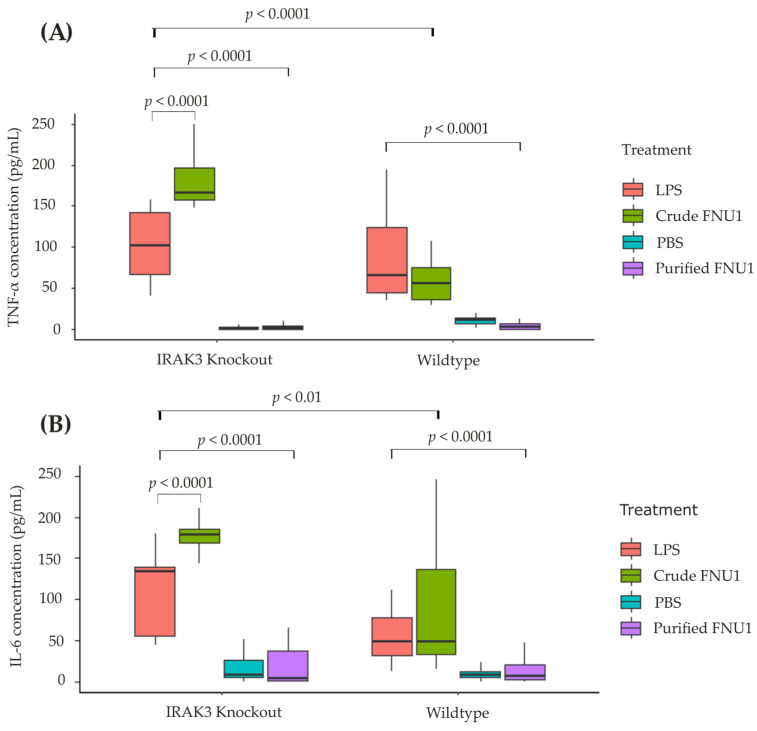
Effects of crude and purified FNU1 on pro-inflammatory cytokine production in IRAK3 knockout and wildtype THP-1 monocytes: (**A**) TNF-α production was significantly higher with LPS and crude FNU1 compared to PBS and purified FNU1 in both wildtype and IRAK3 knockout cells, *p* < 0.0001. (**B**) IL-6 production was significantly higher in both IRAK3 knockout and wildtype monocytes treated with LPS and crude FNU1 compared to PBS and purified FNU1 (*p* < 0.0001).

To determine the innate immune response to FNU1, and the role of the immune modulator IRAK3 in this response, wildtype and IRAK3 knockout THP-1 monocytes were treated with crude and purified FNU1. The effect of these treatments on the production of inflammatory cytokines, TNF-α and IL-6, differed significantly in wildtype and IRAK3 knockout cells (Figure 1). In the wildtype monocytes, production of TNF-α was significantly higher after crude FNU1 exposure with a median (Q1–Q3) of 55.51 (36.47–75.58) pg/mL compared to purified FNU1 with median (Q1–Q3) of 2.34 (0.23–7.00) pg/mL (*p* < 0.0001). Similarly, in IRAK3 knockout monocytes, crude FNU1 treatment produced higher levels (*p* < 0.0001) of TNF-α than purified FNU1 with a median (Q1–Q3) of 172.13 (161.79–246.16) pg/mL and 1.06 (0.13–3.75) pg/mL, respectively (Figure 1A). As with TNF-α, the median (Q1–Q3) production of IL-6 by wildtype cells was significantly higher when treated with crude FNU1 of 48.69 (32.57–137.03) pg/mL than with purified FNU1 at 6.10 (2.80–20.89) pg/mL (*p* < 0.0001). The results for IL-6 production by IRAK3 knockout cells were consistent with those of the wildtype monocytes, showing higher IL-6 levels when treated with crude FNU1 of 178.51 (168.34–185.42) pg/mL than purified FNU1 at 3.49 (1.58–37.83) pg/mL (*p* < 0.0001). The production of both cytokines was elevated in IRAK3 knockout cells compared to the wildtype monocytes after exposure to LPS and crude FNU1 (*p* = 0.01 for IL-6; *p* = 0.0001 for TNF-α). Finally, in the IRAK3 knockout cells, the cytokine responses to crude FNU1 were significantly higher than those to LPS (*p* < 0.001), while in the wildtype cells, there were no differences in TNF-α and IL-6 cytokines (*p* = 0.24 and *p* = 0.32, respectively) (Figure 1). All data are shown in Supplementary Tables S1 and S2. 

**Figure 2:**
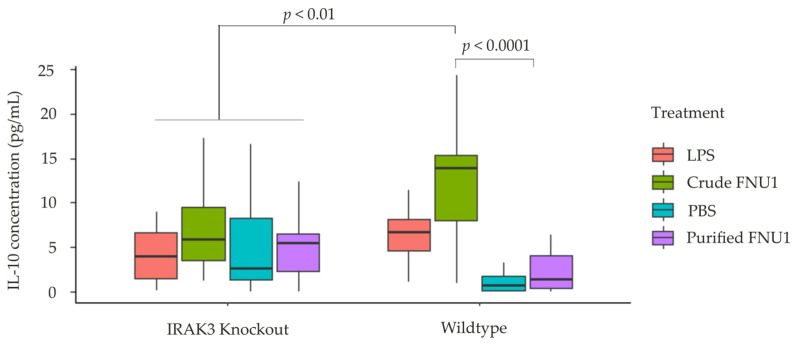
Effects of crude and purified FNU1 on anti-inflammatory cytokine IL-10 production in IRAK3 knockout and wildtype THP-1 monocytes. No difference was seen between treatments in IRAK3 knockout cells; significantly higher IL-10 production was seen in wildtype cells when treated with crude FNU1 or LPS, compared to purified FNU1 or PBS, or when compared to treatments in IRAK3 knockout cells.

Anti-inflammatory cytokines may be expressed to dampen the effects of excessive production of pro-inflammatory cytokines, and this process may be influenced by functional IRAK3 as it is an inhibitory protein in the TLR4 pathway [22]. Thus, the effects of crude and purified FNU1 on the production of the anti-inflammatory cytokine IL-10 by THP-1 wildtype and IRAK3 knockout cells were determined. In wildtype monocytes with functional IRAK3, IL-10 concentrations were significantly higher when exposed to crude bacteriophage compared to when treated with purified FNU1 (median (Q1–Q3) of 13.8 (8.02–15.4) pg/mL and 1.30 (0.43–4.00) pg/mL, respectively, *p* < 0.0001). On the other hand, concentrations of IL-10 were not significantly different in the LPS, crude FNU1, PBS and purified FNU1-treated IRAK3 knockout monocytes (Figure 2). Expression of the pro-inflammatory cytokines TNF-α and IL-6 was comparable between IRAK3 knockout cells and wildtype monocytes when treated with purified FNU1 or PBS, while crude FNU1 or LPS treatment resulted in elevated production in IRAK3 knockout compared to the wildtype cells (Figure 1). This is different for the anti-inflammatory IL-10 where crude FNU1 produces higher levels in wildtype cells compared to the IRAK3 knockout cells (Figure 2). Summary statistics can be found in Supplementary Table S3.

### 2.2. Endotoxin Levels of Crude and Purified FNU1

**Figure 3:**
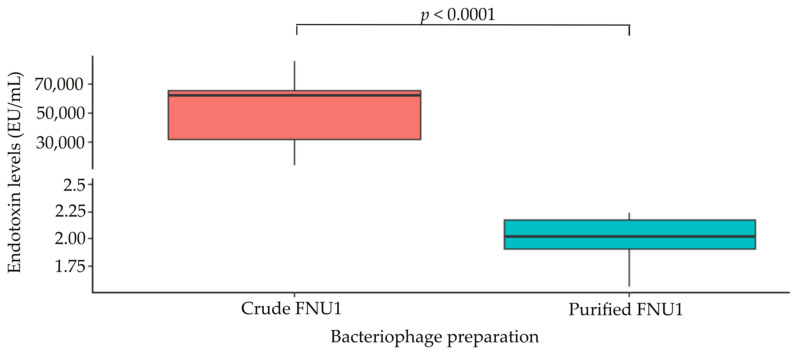
Concentrations of endotoxin present in crude and purified FNU1, as quantified using a commercial endotoxin kit. Crude FNU1 had significantly higher concentrations of endotoxin, expressed as EU/mL, compared to purified samples.

Initially, an amoebocyte endotoxin commercial assay was used to determine the levels of endotoxin, expressed as EU/mL, present in the crude FNU1 and purified FNU1 bacteriophage preparations. Endotoxin levels in crude FNU1 were significantly higher than purified FNU1 (*p* < 0.0001) (Figure 3). Crude FNU1 and purified FNU1 contained a median (Q1–Q3) of 61,632.03 (31,728.33–65,560.06) EU/mL and 2.03 (1.90–2.17) EU/mL of endotoxin, respectively (Supplementary Table S4). 

### 2.3. Development of a Novel Assay to Measure Endotoxin Levels

**Figure 4:**
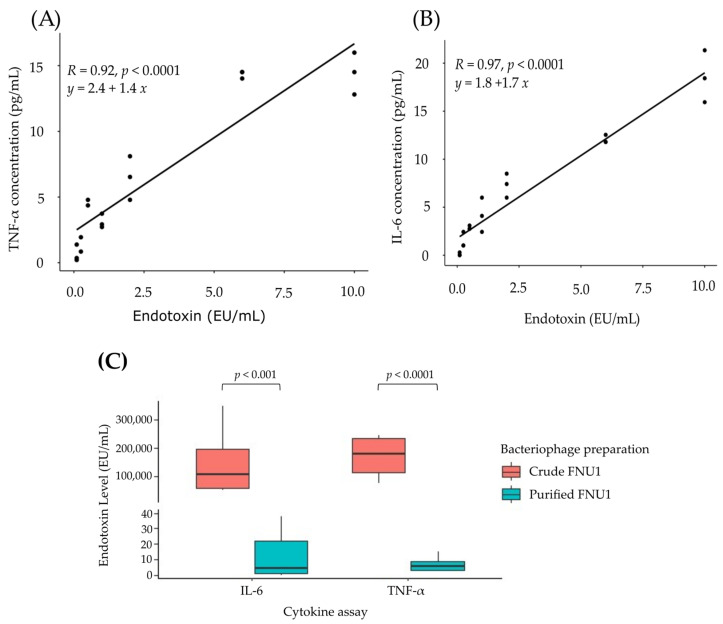
Standard curves of cytokine production by IRAK3 knockout THP-1 cells exposed to known endotoxin concentrations, showing good correlation with levels of endotoxin for expression of (**A**) TNF-α and (**B**) IL-6. (**C**) Using the equation from the standard curves for IL-6 and TNF-α, concentrations of endotoxin in crude FNU1 and purified FNU1 preparations were calculated. Concentrations of endotoxin were significantly higher (*p* < 0.001) in crude FNU1 than purified FNU1 in both IL-6 and TNF-α cytokine assays. There were no differences in levels detected between the two cytokine assays.

A novel assay was developed to estimate endotoxin concentrations. This was achieved by deriving standard curves for TNF-α and IL-6 production in IRAK3 knockout monocytes exposed to known endotoxin amounts (Figure 4). The standard curves showed a linear correlation with the levels of endotoxin (*R*-value close to 1) (Figure 4A,B). IL-6 production resulted in *R* = 0.97 (indicating a 94% chance of predicting endotoxin concentration (*R*^2^ = 0.941)), while for TNF-α, *R* = 0.92 (indicating an 85% chance of predicting endotoxin concentration (*R*^2^ = 0.846)) in the bacteriophage preparations. In wildtype monocytes, there was a lower correlation, compared to IRAK3 knockout cells, between endotoxin and IL-6 cytokine production (*R* = 0.8, *p* < 0.001; *R*^2^ = 0.64, indicating a 64% chance of predicting endotoxin concentrations) or TNF-α production (*R* = 0.52, *p* = 0.02; *R*^2^ = 0.27, indicating a 27% chance of predicting endotoxin concentrations) (Supplementary Figure S1). The IL-6 and TNF-α based assays using IRAK3 knockout cells were, therefore, used to estimate endotoxin concentrations of crude FNU1 and purified FNU1. As determined by these assays, crude FNU1 and purified FNU1 contained significantly different amounts of endotoxin (*p*-value < 0.0001). The median EU/mL (Q1–Q3) for crude FNU1 as measured using TNF-α production was 179,524.57 (115,839.37–234,155.79) EU/mL and 106,543.61 (61,151.40–197,374.35) EU/mL as measured using IL-6 production. (Supplementary Table S5). The purified FNU1 samples had endotoxin concentrations of 4.19 (0.97–22.07) EU/mL as measured using IL-6 production, and 5.36 (8.59–3.00) EU/mL as measured using TNF-α production (Supplementary Table S5). No differences were observed between IL-6 and TNF-α based estimates of endotoxin concentrations (Figure 4C), indicating concordance between the two cytokine-based assays. The levels of endotoxin estimated using the IRAK3 cytokine-based assay were higher than those obtained from the amoebocyte assay.

## 3. Discussion

The precise effects of bacteriophages on the immune system are unclear. Previous reports have suggested that these bacterial viruses may not induce a strong inflammatory reaction [26,27,28]. In this study, it was observed that THP-1 monocyte production of TNF-α and IL-6 was significantly lower in cells exposed to purified FNU1 preparations than in those treated with crude FNU1. The production of TNF-α and IL-6 was significantly higher in the IRAK3 knockout cells than in the wildtype cells, when stimulated by crude FNU1. IRAK3 is an inhibitory protein vital in regulating the overall innate immune response [22,36]. In the IRAK3 knockout cells, the absence of this immune modulator resulted in elevated expression of the pro-inflammatory cytokines. There is limited research on whether bacteriophages exert an anti-inflammatory response through the production of IL-10. One study observed that *Staphylococcus aureus* bacteriophages induced significant expression of IL-10 [29]. In the current study, there was a low level of IL-10 produced in the wildtype THP-1 monocytes in response to purified FNU1, but when the crude FNU1 was added, the IL-10 response was significantly higher. A comparison of the response to the crude FNU1 from the wildtype and IRAK3 knockout monocytes revealed that the absence of IRAK3 modulation in the knockout cells resulted in reduced induction of IL-10 anti-inflammatory cytokine.

A commercial endotoxin detection kit revealed that crude FNU1 contained endotoxin concentrations which were approximately 30,000 times greater than the purified FNU1. However, some of these kits show higher sensitivity than specificity, in that they not only detect intact endotoxin but also degraded molecules (Thermo-Fisher technical department, personal communication). Such degraded molecules may not necessarily affect immune responses. The commercial assay employed here measured responses from non-mammalian cells. Since it is important to understand the effects on human white blood cells, other commercial assays measure endotoxin concentrations based on the reaction of these human cells. However, these kits rely on the production of a single cytokine and have a limited detection range such as measuring only up to 1 EU/mL. Therefore, serial dilutions are required to estimate the concentration of endotoxins in samples, which can be a source of measurement error. Because of these issues, a novel assay was developed to determine endotoxin concentrations of crude and purified FNU1 based on the production of the two main inflammatory cytokines IL-6 and TNF-α when IRAK3 knockout THP-1 monocytes were exposed to a series of known endotoxin concentrations. The data extrapolated from the standard curves showed excellent concordance between results for IL-6 and TNF-α production in response to crude and purified FNU1. These findings indicate the accuracy and robustness of the method devised in this study. Further, the test devised here provides a wider range of endotoxin detection limits up to 10 EU/mL, which is 10 times greater than the commercial endotoxin kits available.

Interestingly, the newly developed assay required using IRAK3 knockout monocytes to establish a linear correlation between TNF-α or IL-6 production versus endotoxin concentrations. Wildtype cells developed plateaus in their cytokine production as endotoxin concentrations increased over 3 EU/mL (Supplementary Figure S1) and consequently have a poor predictive capacity and correlation for higher endotoxin levels. This lack of linear response in the production of IL-6 and TNF-α by wildtype cells as endotoxin concentration rises may be due to the presence of IRAK3 which down-regulates cytokine production. As shown here, in the absence of IRAK3, the levels of TNF-α and IL-6 are not downregulated, and the production of these cytokines increased as the endotoxin levels increased. This data, then, suggests a better correlation between endotoxin levels and cytokine production in THP-1 monocytes that do not have IRAK3-regulated immune responses and as such, our method may be more appropriate in determining endotoxin levels via TNF-α and IL-6 production.

The one-step purification method used in this study yielded similar endotoxin concentrations to those reported previously for extensive bacteriophage purification procedures [37,38]. This finding illustrates that the method used here is efficient and effective as compared to more extensive sequential methods described elsewhere [37]. Further validation of simpler methods to remove endotoxins from bacteriophages is likely to enhance their ability to be used therapeutically especially if combined with rapid, accurate and robust endotoxin detection tests. Several studies have illustrated that purified bacteriophages do not cause an excessive immune response [26,39], but these studies employed different purification methods and distinct bacteriophages. While our work focused on *F. nucleatum* bacteriophage FNU1, other bacteriophages that target diverse species of bacteria should be tested to determine their effects on immune cells and the degree of purification required. Further, the use of in vivo models with varying IRAK3 functionality could assist in clarifying the role of this immune modulator in mediating bacteriophage response, pharmacokinetics, and impact on therapeutic efficacy.

In conclusion, ridding bacteriophages of excess endotoxin is important in reducing immune effects. A robust endotoxin detection assay was developed, using IL-6 and TNF-α response in IRAK3 knockout THP-1 monocytic cells. The novelty and advantage of this assay is that it employs a unique human immune cell line where expression of two of the main inflammatory cytokines correlates well with endotoxin levels, to determine whether bacteriophage preparations are potentially safe for parenteral applications. In addition, the simple bacteriophage purification method employed here is as effective in reducing endotoxin concentrations as those that require more extensive and time-consuming steps.

## 4. Materials and Methods

### 4.1. Monocyte Cell Culture

Wildtype (American Type Culture Collection ATCC, TIB-202) and IRAK-3 knockout THP-1 monocytic cell lines were used in this study as described by Nguyen et al. [25]. THP-1 cells were grown in Roswell Park Memorial Institute (RPMI) 1640 medium with L-Glutamine and HEPES (Gibco—ThermoFisher Scientific, 11875-093, Scoresby, VIC, Australia) and supplemented with 10% foetal bovine serum (FBS; Bovogen Biologicals’, SFBS-AU, Keilor East, VIC, Australia). The cells were incubated in 5% CO_2_ at 37 °C. Using prewarmed growth media (37 °C), the cells were passaged when they had grown to 80% confluency and subsequently split for another three passages. PCR Mycoplasma Detection Kit (TOKU-E, M034, Bellingham, WA, USA) was used to ensure the cells were mycoplasma-free before they were used in the experiments.

### 4.2. Fusobacterium Nucleatum Culture

*F. nucleatum* (ATCC 10953) was grown on heart infusion (HI) agar, containing HI broth (Oxoid, CM1032, Thebarton, SA, Australia), 0.5% haemin (Sigma-Aldrich, 51280, Bayswater, VIC, Australia) and 1% bacteriological agar (Oxoid, LP0011, Thebarton, SA, Australia). The culture was grown in anaerobic conditions using anaerobic generating sachets (AnaeroGen^TM^ 2.5L, AN0025A, Oxoid, Thebarton, SA, Australia) at 37 °C for 48 h.

### 4.3. Purification of F. nucleatum Bacteriophage FNU1

To generate a high concentration (1 × 10^8^ plaque-forming units (PFU) per mL) of FNU1, a 200 µL suspension of bacteriophage FNU1, prepared as described previously [15] was spread onto HI agar with a fresh lawn of *F. nucleatum*. After 48 h of anaerobic incubation at 37 °C, the bacteriophage clearance zones were washed with 3 mL of phosphate-buffered saline pH 7.4 (PBS). This was then centrifuged at 4000× *g*, and supernatant was collected and filtered using 0.2 mm cellulose acetate filters (Advantec, 25CS020AS, Taren Point, NSW, Australia) to collect what we termed the “crude” FNU1 preparation.

The purification method of FNU1 is similar to that previously described [40]. To purify the crude FNU1 of endotoxin, 2 mL of 2.5 M magnesium chloride (MgCl_2_), 1 mL of DNase I (Promega, M6101, Sydney, NSW, Australia) and 1 mL RNase A (Promega, A7973, Australia) were added to 1 mL of bacteriophage suspension. After incubation at room temperature for 30 min, polyethylene glycol (PEG) 8000 (Sigma-Aldrich, P2139, Bayswater, VIC, Australia) and NaCl (Sigma-Aldrich, S-7653, Bayswater, VIC, Australia) were added to a final concentration of 10% *w*/*v* and 1 mM, respectively, and the mixture was gently shaken until solubilised. Triton X-100 (Sigma-Aldrich, T8787, Bayswater, VIC, Australia) was then added to a final concentration of 2% *v*/*v* before incubating at 4 °C for 15 min. Bacteriophages were precipitated by centrifuging at 14,000× *g*, 4 °C, for 10 min. The supernatant was removed and replaced with 1 mL PBS. The steps of exposure to PEG 8000, NaCl, Triton X-100 and precipitation were repeated three times. The bacteriophages were then resuspended in 1 mL PBS. Three washes involving centrifugation at 14,000× *g*, 4 °C for 10 min, and resuspension of the pellet in PBS were performed. The bacteriophage suspension was then filtered through 0.2 mm cellulose acetate filters (Advantec, 25CS020AS, Taren Point, NSW, Australia), and this constituted what we termed the “purified” FNU1 preparation.

### 4.4. Cytokine Assays

Wildtype and IRAK-3 knockout THP-1 cells were split into approximately 2 × 10^5^ cells per well in 12-well plates. The cells were treated with crude or purified bacteriophage at 3.0 × 10^8^ PFU/mL. PBS was used as a negative control and 1 mg/mL of LPS (*Escherichia coli* O55:B5, Sigma-Aldrich, L2880, Bayswater, VIC, Australia)) as the positive control; 300 mL of each treatment was added to the cells, and the cells were incubated for 24 h, followed by centrifugation (300× *g*, 5 min, 37 °C). The supernatant was collected, and then Enzyme-Linked Immunosorbent Assays (ELISA), for TNF-α, IL-6 and IL-10 (BD OptEIA™ Human ELISA Set; TNF-α, 555212; IL-6, 555220; IL-10, 555157, North Ryde, VIC, Australia) were performed, according to manufacturer’s instructions. Data were collected from eight replicates in each of the three independent experiments. A standard curve was generated for each cytokine and the production of cytokines was determined from this.

### 4.5. Endotoxin Detection Assay

Levels of endotoxin in crude and purified FNU1 preparations were tested using an amoebocyte-based assay (Pierce Chromogenic Endotoxin Quant Kit, ThermoFisher Scientific, A39552S, Scoresby, VIC, Australia). The amoebocyte assay was performed according to the manufacturer’s instructions, and a standard curve was generated. Samples were diluted to ensure that they fell within the linear portion of the standard curve enabling endotoxin concentration (as EU/mL) to be determined. Data were collected from three independent experiments, with four replicates in each (*n* = 12).

### 4.6. Developing Novel Endotoxin Quantification Assay

Wildtype and IRAK3 knockout THP-1 cells were treated as described above with known concentrations (10, 6, 2, 1, 0.5, 0.25, 0.1, 0 EU/mL) of endotoxin standards (Pierce Chromogenic Endotoxin Quant Kit). Cytokine production was measured using ELISA assays for TNF-α and IL-6 as described above. Data were obtained from three independent experiments, each with 4 replicates (*n* = 12). Endotoxin units per mL (EU/mL) were plotted against cytokine levels to generate a standard curve that was used to estimate the amount of endotoxin present in crude and purified FNU1.

### 4.7. Statistical Analysis

All data were collected into Excel spreadsheets before importing to R/R studio. The Shapiro–Wilk test was used to determine whether data on the cytokine production and endotoxin levels were normally distributed. As the data were not normally distributed, they were analysed in terms of median rather than means and were represented as boxplots using the ggboxplot function in the ggplot2 package in R. The ggpubr package was used to plot the standard curves of EU/mL against cytokine levels. Scatter plots were made using the ggscatter function, the stat_cor and stat_regline_equation functions were used to calculate the correlation coefficient (*R*) and equation of the linear regression line. Wilcoxon signed rank, a non-parametric statistical test, was used to compare the medians in cytokine and endotoxin levels between different treatments. A *p*-value less than 0.05 was considered statistically significant. All analyses were completed through R/Rstudio software (R version 4.3.0 (2023.06.0 + 421)).

## Data Availability

Bacteriophage FNU1 genome sequence is available on NCBI GenBank accession No: MK554696. All other data is presented here and available in Supplementary Data.

## Supplementary Materials

The supporting information can be downloaded at: https://www.mdpi.com/article/10.3390/ijms242015108/s1.

## References

[1] Moore W.E., Moore L.V. (1994). The bacteria of periodontal diseases. Periodontology 2000.

[2] Brennan C.A., Garrett W.S. (2019). *Fusobacterium nucleatum*—Symbiont, opportunist and oncobacterium. Nat. Rev. Microbiol..

[3] Castellarin M., Warren R.L., Freeman J.D., Dreolini L., Krzywinski M., Strauss J., Barnes R., Watson P., Allen-Vercoe E., Moore R.A. (2012). *Fusobacterium nucleatum* infection is prevalent in human colorectal carcinoma. Genome Res..

[4] Abed J., Maalouf N., Manson A.L., Earl A.M., Parhi L., Emgard J.E.M., Klutstein M., Tayeb S., Almogy G., Atlan K.A. (2020). Colon Cancer-Associated *Fusobacterium nucleatum* May Originate From the Oral Cavity and Reach Colon Tumors via the Circulatory System. Front. Cell. Infect. Microbiol..

[5] Kostic A.D., Chun E., Robertson L., Glickman J.N., Gallini C.A., Michaud M., Clancy T.E., Chung D.C., Lochhead P., Hold G.L. (2013). *Fusobacterium nucleatum* potentiates intestinal tumorigenesis and modulates the tumor-immune microenvironment. Cell Host Microbe.

[6] Han Y.W. (2015). *Fusobacterium nucleatum*: A commensal-turned pathogen. Curr. Opin. Microbiol..

[7] Laxminarayan R. (2022). The overlooked pandemic of antimicrobial resistance. Lancet.

[8] Hanlon G.W. (2007). Bacteriophages: An appraisal of their role in the treatment of bacterial infections. Int. J. Antimicrob. Agents.

[9] Loc-Carrillo C., Abedon S.T. (2011). Pros and cons of phage therapy. Bacteriophage.

[10] Dicks L.M.T., Mikkelsen L.S., Brandsborg E., Marcotte H. (2019). *Clostridium difficile*, the Difficult “Kloster” Fuelled by Antibiotics. Curr. Microbiol..

[11] Theochari N.A., Stefanopoulos A., Mylonas K.S., Economopoulos K.P. (2018). Antibiotics exposure and risk of inflammatory bowel disease: A systematic review. Scand. J. Gastroenterol..

[12] Boursi B., Mamtani R., Haynes K., Yang Y.X. (2015). Recurrent antibiotic exposure may promote cancer formation-Another step in understanding the role of the human microbiota?. Eur. J. Cancer.

[13] Nir-Paz R., Gelman D., Khouri A., Sisson B.M., Fackler J., Alkalay-Oren S., Khalifa L., Rimon A., Yerushalmy O., Bader R. (2019). Successful treatment of antibiotic resistant poly-microbial bone infection with bacteriophages and antibiotics combination. Clin. Infect. Dis..

[14] Lusiak-Szelachowska M., Weber-Dabrowska B., Gorski A. (2020). Bacteriophages and Lysins in Biofilm Control. Virol. Sin..

[15] Kabwe M., Brown T.L., Dashper S., Speirs L., Ku H., Petrovski S., Chan H.T., Lock P., Tucci J. (2019). Genomic, morphological and functional characterisation of novel bacteriophage FNU1 capable of disrupting *Fusobacterium nucleatum* biofilms. Sci. Rep..

[16] Kropinski A.M., Tolstoy I., Adriaenssens E.M., Kabwe M., Tucci J. (2020). Taxon Details: Latrobevirus FNU1. https://ictv.global/taxonomy/taxondetails?taxnode_id=202210207.

[17] Szermer-Olearnik B., Boratyński J. (2015). Removal of endotoxins from bacteriophage preparations by extraction with organic solvents. PLoS ONE.

[18] Martich G.D., Boujoukos A.J., Suffredini A.F. (1993). Response of man to endotoxin. Immunobiology.

[19] Podlacha M., Grabowski Ł., Kosznik-Kawśnicka K., Zdrojewska K., Stasiłojć M., Węgrzyn G., Węgrzyn A. (2021). Interactions of bacteriophages with animal and human organisms—Safety issues in the light of phage therapy. Int. J. Mol..

[20] Rosadini C.V., Kagan J.C. (2017). Early innate immune responses to bacterial LPS. Curr. Opin. Allergy Clin. Immunol..

[21] Du J., Nicolaes G.A., Kruijswijk D., Versloot M., van der Poll T., van’t Veer C. (2014). The structure function of the death domain of human IRAK-M. Cell Commun. Signal..

[22] Kobayashi K., Hernandez L.D., Galán J.E., Janeway C.A., Medzhitov R., Flavell R.A. (2002). IRAK-M is a negative regulator of Toll-like receptor signaling. Cell.

[23] Freihat L.A., Wheeler J.I., Wong A., Turek I., Manallack D.T., Irving H.R. (2019). IRAK3 modulates downstream innate immune signalling through its guanylate cyclase activity. Sci. Rep..

[24] Turek I., Nguyen T.H., Galea C., Abad I., Freihat L., Manallack D.T., Velkov T., Irving H. (2023). Mutations in the Vicinity of the IRAK3 Guanylate Cyclase Center Impact Its Subcellular Localization and Ability to Modulate Inflammatory Signaling in Immortalized Cell Lines. Int. J. Mol. Sci..

[25] Nguyen T.H., Axell A., Turek I., Wright B., Meehan-Andrews T., Irving H.R. (2022). Modulation of Inflammatory Cytokine Production in Human Monocytes by cGMP and IRAK3. Int. J. Mol. Sci..

[26] Miernikiewicz P., Dabrowska K., Piotrowicz A., Owczarek B., Wojas-Turek J., Kicielinska J., Rossowska J., Pajtasz-Piasecka E., Hodyra K., Macegoniuk K. (2013). T4 phage and its head surface proteins do not stimulate inflammatory mediator production. PLoS ONE.

[27] Møller-Olsen C., Ross T., Leppard K.N., Foisor V., Smith C., Grammatopoulos D.K., Sagona A.P. (2020). Bacteriophage K1F targets *Escherichia coli* K1 in cerebral endothelial cells and influences the barrier function. Sci. Rep..

[28] Weber-Dabrowska B., Zimecki M., Mulczyk M. (2000). Effective phage therapy is associated with normalization of cytokine production by blood cell cultures. Arch. Immunol. Ther. Exp..

[29] Zhang L., Hou X., Sun L., He T., Wei R., Pang M., Wang R. (2018). *Staphylococcus aureus* Bacteriophage Suppresses LPS-Induced Inflammation in MAC-T Bovine Mammary Epithelial Cells. Front. Microbiol..

[30] Van Belleghem J.D., Clement F., Merabishvili M., Lavigne R., Vaneechoutte M. (2017). Pro- and anti-inflammatory responses of peripheral blood mononuclear cells induced by *Staphylococcus aureus* and *Pseudomonas aeruginosa* phages. Sci. Rep..

[31] Marongiu L., Burkard M., Lauer U.M., Hoelzle L.E., Venturelli S. (2022). Reassessment of Historical Clinical Trials Supports the Effectiveness of Phage Therapy. Clin. Microbiol. Rev..

[32] Stacey H.J., De Soir S., Jones J.D. (2022). The Safety and Efficacy of Phage Therapy: A Systematic Review of Clinical and Safety Trials. Antibiotics.

[33] Dedrick R.M., Freeman K.G., Nguyen J.A., Bahadirli-Talbott A., Smith B.E., Wu A.E., Ong A.S., Lin C.T., Ruppel L.C., Parrish N.M. (2021). Potent antibody-mediated neutralization limits bacteriophage treatment of a pulmonary Mycobacterium abscessus infection. Nat. Med..

[34] Wu N., Dai J., Guo M., Li J., Zhou X., Li F., Gao Y., Qu H., Lu H., Jin J. (2021). Pre-optimized phage therapy on secondary Acinetobacter baumannii infection in four critical COVID-19 patients. Emerg. Microbes Infect..

[35] Food and Drug Administration (2020). Setting Endotoxin Limits during Development of Investigational Oncology Drugs and Biological Products Guidance for Industry.

[36] Nguyen T.H., Turek I., Meehan-Andrews T., Zacharias A., Irving H.R. (2022). A systematic review and meta-analyses of interleukin-1 receptor associated kinase 3 (IRAK3) action on inflammation in in vivo models for the study of sepsis. PLoS ONE.

[37] Luong T., Salabarria A.-C., Edwards R.A., Roach D.R. (2020). Standardized bacteriophage purification for personalized phage therapy. Nat. Protoc..

[38] Van Belleghem J.D., Merabishvili M., Vergauwen B., Lavigne R., Vaneechoutte M. (2017). A comparative study of different strategies for removal of endotoxins from bacteriophage preparations. J. Microbiol. Methods.

[39] Schubert B.D., Ku H., Kabwe M., Nguyen T.H., Irving H., Tucci J. (2022). Effects of *Klebsiella pneumoniae* Bacteriophages on IRAK3 Knockdown/Knockout THP-1 Monocyte Cell Lines. Viruses.

[40] Branston S.D., Wright J., Keshavarz-Moore E. (2015). A non-chromatographic method for the removal of endotoxins from bacteriophages. Biotechnol. Bioeng..

