# Supplementary material for: IRAK3 Knockout and Wildtype THP-1 Monocytes as Models for Endotoxin Detection Assays and Fusobacterium nucleatum Bacteriophage FNU1 Cytokine Induction
Source: Int J Mol Sci. 2023 Oct 12;24(20):15108. doi: 10.3390/ijms242015108 (PMC10606876; doi:10.3390/ijms242015108)
Supplement: Supplementary file 1 [file ijms-24-15108-s001.zip › ijms-2602541-supplementary.pdf]

International Journal of
*Molecular Sciences*

MDPI

**Supplementary materials**

**Table S1. Production of TNF-$\alpha$ by THP-1 wildtype (WT) and IRAK3 knockout monocytes**

| | Treatment | TNF-$\alpha$ concentration (pg/mL) | | | | |
|---|---|---|---|---|---|---|
| | | Minimum | 25th percentile | Median | 75th percentile | Maximum |
| 1 | PBS (WT) | 0.67 | 6.71 | 10.48 | 13.84 | 18.94 |
| 2 | Purified FNU1 (WT) | 0.00 | 0.23 | 2.34 | 7.00 | 12.49 |
| 3 | LPS (WT) | 34.21 | 44.58 | 64.92 | 123.51 | 193.80 |
| 4 | Crude FNU1 (WT) | 29.08 | 36.47 | 55.51 | 75.58 | 107.43 |
| 5 | PBS (IRAK3 Knockout) | 0.00 | 0.17 | 0.89 | 2.28 | 5.35 |
| 6 | Purified FNU1 (IRAK3 Knockout) | 0.00 | 0.13 | 1.06 | 3.75 | 9.13 |
| 7 | LPS (IRAK3 Knockout) | 40.47 | 66.15 | 100.95 | 142.48 | 157.89 |
| 8 | Crude FNU1 (IRAK3 Knockout) | 147.91 | 161.79 | 172.13 | 246.16 | 267.90 |

**Table S2. Production of IL-6 by wildtype (WT) and IRAK3 knockout THP-1 monocytes**

| | Treatment | IL-6 concentration (pg/mL) | | | | |
|---|---|---|---|---|---|---|
| | | Minimum | 25th percentile | Median | 75th percentile | Maximum |
| 1 | PBS (WT) | 0.00 | 5.13 | 8.54 | 12.70 | 22.76 |
| 2 | Purified FNU1 (WT) | 0.00 | 2.80 | 6.10 | 20.89 | 47.47 |
| 3 | LPS (WT) | 11.55 | 32.03 | 48.12 | 77.52 | 111.36 |
| 4 | Crude FNU1 (WT) | 15.21 | 32.57 | 48.69 | 137.03 | 246.05 |
| 5 | PBS (IRAK3 Knockout) | 0.00 | 4.48 | 8.01 | 26.62 | 51.11 |
| 6 | Purified FNU1 (IRAK3 Knockout) | 0.00 | 1.58 | 3.49 | 37.83 | 65.90 |
| 7 | LPS (IRAK3 Knockout) | 44.35 | 55.54 | 133.35 | 139.49 | 179.78 |
| 8 | Crude FNU1 (IRAK3 Knockout) | 143.05 | 168.34 | 178.51 | 185.42 | 210.77 |

**Table S3. Production of IL-10 by wildtype (WT) and IRAK3 knockout THP-1 monocytes**

| | | IL-10 concentration (pg/mL) | | | | |
|---|---|---|---|---|---|---|
| Monocyte type | Treatment | Minimum | 25th percentile | Median | 75th percentile | Maximum |
| Wildtype | PBS | 0 | 0.14 | 0.68 | 1.725 | 3.28 |
| | Purified FNU1 | 0 | 0.43 | 1.295 | 3.9975 | 6.35 |
| | LPS | 1.05 | 4.525 | 6.64 | 8.105 | 11.34 |
| | Crude FNU1 | 0.96 | 8.02 | 13.8 | 15.38 | 24.39 |
| IRAK3 knockout | PBS | 0 | 1.38 | 2.49 | 8.29 | 16.52 |
| | Purified FNU1 | 0 | 2.2625 | 5.37 | 6.475 | 12.31 |
| | LPS | 0.11 | 1.5175 | 3.945 | 6.66 | 8.94 |
| | Crude FNU1 | 1.15 | 3.44 | 5.83 | 9.55 | 17.25 |

**Table S4. Levels of endotoxins present in purified and crude bacteriophage FNU1 quantified using an endotoxin detection kit**

| Bacteriophage preparation | Levels of endotoxin (EU/mL) | | | | |
|---|---|---|---|---|---|
| | Minimum | 25th percentile | Median | 75th percentile | Maximum |
| Crude FNU1 | 13988.85 | 31728.33 | 61632.03 | 65560.06 | 85453.62 |
| Purified FNU1 | 1.55 | 1.90 | 2.02 | 2.17 | 2.24 |

**Table S5. Estimated levels of endotoxin present in THP-1 knockout out samples after crude and purified FNU1 treatment using the new endotoxin assay**

| Cytokine | Bacteriophage | Levels of endotoxin (EU/mL) | | | | |
|---|---|---|---|---|---|---|
| | Preparation | Minimum | 25th percentile | Median | 75th percentile | Maximum |
| IL-6 | Crude FNU1 | 52970.57 | 61151.40 | 106543.61 | 197374.35 | 347747.78 |
| | Purified FNU1 | 0.13 | 0.97 | 4.19 | 22.07 | 37.71 |
| TNF-$\alpha$ | Crude FNU1 | 78376.38 | 115839.37 | 179524.57 | 234155.79 | 244456.83 |
| | Purified FNU1 | 2.40 | 3.00 | 5.36 | 8.59 | 15.18 |

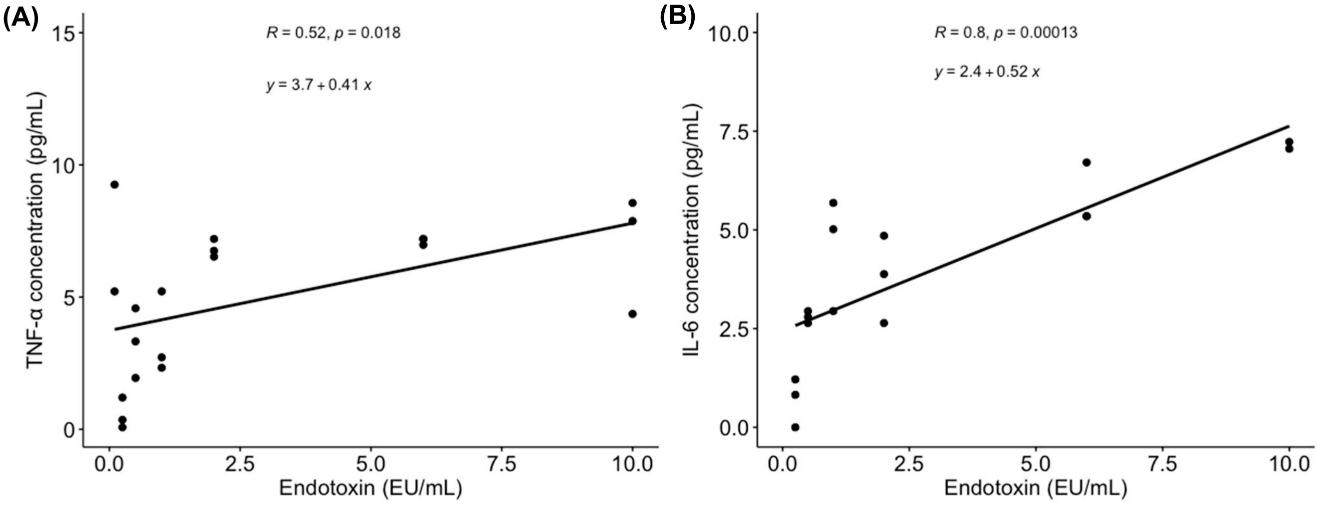


Figure S1. Standard curve of cytokine production by THP-1 wildtype monocytes against endotoxin levels; (A) TNF-$\alpha$ indicate an $R = 0.52$ and 27% chance of predicting endotoxin levels from an $R^2$ of 0.27, while (B) IL-6 indicate an $R = 0.8$ and 64% chance of predicting endotoxin levels from $R^2 = 0.64$.

Known endotoxin concentrations (10, 6, 2, 1, 0.5, 0.25, 0.1, 0 EU/mL) were incubated with THP-1 monocytes over 24 hours. Cell supernatant was collected and analysed for cytokine production by ELISA.
